# Quality Improvement Curriculum for Intensive Care Unit Upgrades

**DOI:** 10.5811/westjem.59400

**Published:** 2023-10-11

**Authors:** Seth R. Bohman, Lauren Day, Colin Danko, Bhaskar Thakur, Raashee Kedia, Samuel Parnell

**Affiliations:** * University of Texas Southwestern Medical Center, Department of Emergency Medicine, Dallas, Texas; † University of Texas Southwestern Medical Center, Department of Population and Data Science, Dallas, Texas

## Abstract

Patients admitted to the hospital ward from the emergency department (ED) occasionally decompensate and require transfer to the intensive care unit (ICU). An emergency medicine (EM) curriculum focused on review of these ICU upgrade cases could improve resident knowledge related to patient acuity, critical illness, and appropriate disposition. Furthermore, initial identification of critical pathology in the ED and earlier admission to the ICU could reduce delays in care and improve patient outcomes.

We performed a retrospective analysis to determine the effectiveness of a resident quality improvement curriculum evaluating cases where patients require transfer from the inpatient floor to the ICU within 12 hours of admission from the ED. We compared postgraduate year 2 (PGY-2) EM residents who participated in the ICU upgrades curriculum during their first year to PGY-2 EM residents who did not participate in the curriculum.

Analysis of the 242 qualifying ICU upgrade cases from July 2019–October 2021 showed post-curriculum residents were responsible for an average of 1.0 upgrades per resident compared to an average of 1.54 upgrades per resident (*P* = 0.12) for pre-curriculum residents. Although there was no statistically significant difference in ICU upgrades between the groups, there was a trend toward decreased ICU upgrade cases for residents who participated in the curriculum. Common reasons for ICU upgrade included worsening respiratory distress requiring higher level of respiratory support, recurrent hypotension after initial intravenous fluid resuscitation requiring vasopressor support, and declining mental status.

This retrospective study showed no significant difference in the number of ICU upgrades for residents who completed the ICU upgrades curriculum compared to residents who were not enrolled in the course. However, the study was likely underpowered to detect a significant difference in the groups, and there was a trend toward reduced ICU upgrades for residents who completed the curriculum. ICU upgrade cases were frequently associated with worsening respiratory status, hypotension, and mental status. These findings highlight the importance of reassessment of vital signs and mental status prior to determining disposition from the ED. Additional, larger studies are needed to better determine the curriculum’s impact on resident proficiency in recognizing critical illness and reducing ICU upgrades.

Population Health Research Capsule What do we already know about this issue?

*Patients admitted from the ED occasionally decompensate and require transfer to the ICU. These upgrades to ICU care can be associated with delayed care and worse outcomes.*

What was the research questions?

*What was the impact of a resident quality improvement course on the number of ICU upgrades within 12 hours of admission from the ED?*

What was the major finding of the study?

*Post-course residents averaged 1.0 upgrades/resident vs 1.54 upgrades/resident (P = 0.12) for pre-course residents.*

How does this improve population health?

*While we did not detect a significant difference between groups, there was a trend toward reduced ICU upgrades for residents who completed the course.*

## BACKGROUND

Emergency physicians care for undifferentiated patients with a wide range of acuity. Making the correct diagnosis and determining the appropriate disposition can be challenging, especially for resident physicians in training. However, appropriately determining patient disposition, such as discharge vs floor admission vs intensive care unit (ICU) admission, is one of the most important roles of an emergency physician. Patients initially triaged as stable for hospital ward admission occasionally decompensate and require rapid upgrade in care to the ICU setting. These ICU upgrades can lead to disjointed and delayed patient care, inefficient resource utilization, and undesirable outcomes for the patient, clinician, and healthcare system.^1^ ^,^ ^2^ ^,^ ^3^

Initial identification of critical pathology in the emergency department (ED) and earlier admission to the ICU could reduce delays in care and improve patient outcomes. A residency quality improvement (QI) curriculum focused on reviewing these ICU upgrade cases could improve resident proficiency in determining appropriate patient disposition, reduce the number of ICU upgrades, and enhance the quality of patient care.

## OBJECTIVES

The purpose of this educational initiative was to create a QI curriculum focused on structured case reviews and root cause analyses for patients who were initially admitted from the ED to the inpatient floor and subsequently required transfer to the ICU within 12 hours of admission. We chose a 12-hour window by consensus opinion, as this was considered a reasonable time frame in which clinical deterioration might be anticipated and not so long as to be significantly impacted by floor interventions or lack thereof. The curriculum was implemented at a large, urban, academic tertiary-care facility with an established emergency medicine (EM) residency program. After implementation, we performed a retrospective, observational analysis of the educational initiative, comparing the incidence rate ratio of ICU upgrade cases for postgraduate year 2 (PGY-2) post-curriculum EM residents to PGY-2 pre-curriculum EM residents.

## CURRICULAR DESIGN

The UT Southwestern Medical Center Emergency Medicine Residency Program developed a QI curriculum called Residents Enhancing Safety and Quality (RES-Q). This curriculum allows residents the opportunity to rotate through different QI-focused subgroups every six months. The current project addresses the ICU-upgrades aspect of this curriculum and the impact of this program on the likelihood of resident physicians being involved in ICU upgrade cases.

Each ICU upgrade was identified in the electronic health record (EHR) system with the help of EHR query tools and information technology staff. We excluded cases if the patient went directly from the ED to the operating room, if the patient had an ICU specialty consult in the ED prior to admission, or if the patient was cared for by only an attending physician or an advanced practice practitioner.

At the beginning of each month, the residents in the ICU Upgrades RES-Q group received a report with all the ICU upgrade cases from the previous month. The upgrade cases were then divided among the residents in the RES-Q group. Residents were instructed to thoroughly review all notes and documentation related to each ICU upgrade case, including clinician notes, nursing notes, diagnostic study results, vital signs, and medication administration reports. Residents were tasked with identifying any indications of the patients’ impending decompensation during their time in the ED and potential root causes for the upgrade to ICU care.^4^

Finally, the residents made note of any opportunities for improvement in diagnosis or management that could have affected the clinical course and possibly negated the need for an ICU upgrade. Faculty were available to discuss the cases and possible learning points, but the exercise was primarily resident driven. Cases that were deemed to be of high learning potential by the faculty were subsequently presented to the entire residency program during weekly academic conference.

We submitted this study as an educational process improvement project. It was reviewed by a QI committee at UT Southwestern, and institutional review board approval was deemed unnecessary.

## IMPACT/EFFECTIVENESS

After the RES-Q ICU upgrades curriculum was implemented, we performed a retrospective, observational analysis. This study took place at a large, urban, academic tertiary-care facility in Dallas, Texas, associated with an EM residency program. The duration of the study was July 2019–October 2021.

The primary outcome of the study was a quantification of the number of cases in which patients seen by PGY-2 EM residents required an upgrade to ICU care. We chose PGY-2 residents to reduce the variability in clinical experience that would result from including residents of all academic years. By comparing residents of the same year randomly assigned to complete the ICU upgrades curriculum, confounding variables were minimized.

Our analysis compared PGY-2 EM residents who participated in the ICU upgrades curriculum their first year to PGY-2 EM residents who did not participate in the curriculum their first year. We then estimated the method of maximum likelihood by fitting a generalized Poisson linear regression model to the data.

**Figure 1.**
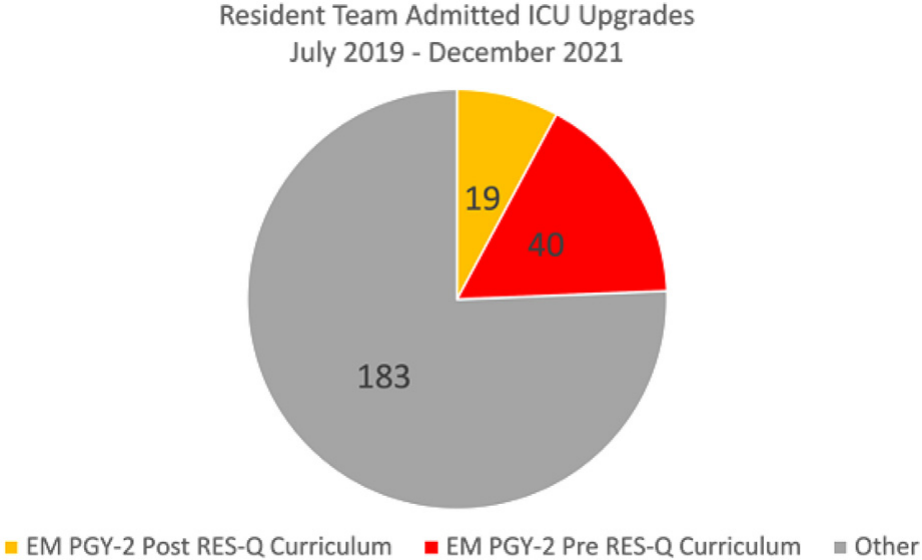
Breakdown of upgrades to intensive care unit care. *EM*, emergency medicine; *PGY*, postgraduate year; *RES-Q*, Residents Enhancing Safety and Quality.

Analysis of the 242 qualifying resident ICU upgrade cases from July 2019–October 2021 showed that 19 PGY-2 EM residents who completed the curriculum were responsible for 19 ICU upgrades, and 26 PGY-2 EM residents who had not yet completed the curriculum were responsible for 40 ICU upgrades. The incidence rate ratio of ICU upgrade cases for PGY-2 pre-curriculum residents was 1.54 (95% confidence interval 0.89–2.66; *P* = 0.122) compared to PGY-2 post-curriculum residents. See Figure 1 for a breakdown of the number of ICU upgrade cases.

Although we found no statistically significant difference in ICU upgrades between the groups, there was a trend toward decreased ICU upgrade cases for residents who participated in the curriculum. Over the study period, residents who completed the ICU upgrades curriculum had a 35% relative risk reduction in ICU upgrades compared to their pre-curriculum colleagues.

During review of ICU upgrade cases, we identified several circumstances associated with an ICU upgrade. Common reasons for transfer from the floor to the ICU after initial ED evaluation included worsening respiratory distress requiring intubation or higher level of respiratory support; recurrent hypotension after initial intravenous (IV) fluid resuscitation requiring vasopressor support; and declining mental status. Specifically, those patients who needed high-flow nasal cannula or non-invasive ventilation for respiratory support and those who required multiple liters of IV fluids for hypotension were at high risk for subsequent ICU upgrade. Another common reason for ICU upgrade was development or worsening of alcohol withdrawal. These common reasons for ICU upgrade suggest that deteriorating clinical status from initial ED evaluation is a frequent root cause of ICU upgrades. These cases highlight the importance of frequent patient reassessment prior to determining final disposition.

Although not statistically significant, this data is promising. A simple educational intervention with minimal cost to the healthcare system was potentially associated with reduced patient transfers from the floor to the ICU. Similar QI programs could improve resident training in identifying critical illness and potentially lead to improved patient outcomes, more appropriate resource utilization, and decreased healthcare costs. Additional time periods and residency classes are currently under review to better determine the effect of the RES-Q ICU upgrades curriculum.

## LIMITATIONS

This study had several limitations. This was a retrospective, observational study that was conducted at a single academic medical center. The smaller sample size specifically reduced the power of the study and decreased the likelihood of detecting a significant difference between the groups. In addition, although only the residents in the RES-Q group went through the structured case review of ICU upgrades, all residents at the program were exposed to teaching points from the monthly RES-Q conference lectures. We were not able to control for the attending on shift or other unidentified factors that may have taken the medical decision-making responsibility away from the resident. We did not account for patient volume or ED boarding of inpatient admissions, which could have influenced length of stay and impacted the number of ICU upgrades. Patient and resident demographic data was not collected during this study, which could be an area of subsequent research. Future studies could investigate the effect of the RES-Q ICU upgrades QI curriculum at other EM programs. This would increase the sample size and provide external validity across other programs and patient populations.

## CONCLUSION

This study demonstrated that completion of the RES-Q ICU upgrades curriculum was not associated with a significant difference in the number of patients who required transfer from the inpatient floor to the ICU within 12 hours of admission. However, completion of the quality improvement curriculum was associated with a trend toward decreased ICU upgrades. The ICU upgrade cases were frequently associated with worsening respiratory status, hypotension, and mental status. These findings highlight the importance of reassessment of vital signs and mental status prior to determining disposition from the ED. Additional, larger studies are needed to determine whether the curriculum has a significant impact on ICU upgrades and can improve resident proficiency in recognizing critical illness and appropriately triaging the clinical acuity of patients. With tools in the electronic health record and appropriate buy-in from residents and program leadership, this curriculum could be easily replicated at other EM residency training programs.
